# Event Report on the 5^th^ Teaching Course on Rare Neurological Diseases

**DOI:** 10.25122/jml-2022-1026

**Published:** 2022-09

**Authors:** Stefana-Andrada Dobran, Alexandra-Mihaela Gherman, Dafin Fior Muresanu

**Affiliations:** 1 RoNeuro Institute for Neurological Research and Diagnostic, Cluj-Napoca, Romania; 2 Sociology Department, Babes-Bolyai University, Cluj-Napoca, Romania; 3 Department of Neuroscience, Iuliu Hatieganu University of Medicine and Pharmacy, Cluj-Napoca, Romania

The 5^th^ Teaching Course on Rare Neurological Diseases took place on the 12^th^ of July 2022, following the last day of the 2022 17^th^ International Summer School of Neurology. The inspiring digital event brought together international speakers and presented a multidisciplinary approach to one of the less-addressed topics in neurology. The event it was coordinated by **Antonio Federico (Italy)**, Professor of Neurology at the Department of Medicine, Surgery and Neurosciences from the Medical School at the University of Siena (Italy), and **Dafin Muresanu (Romania)**, EFNR President, and was divided into 4 sessions over the course of the day.

The Teaching Course on Rare Neurological Diseases began with a welcome address by **Antonio Federico (Italy)** and **Dafin Muresanu (Romania)**, with the two highlighting the increased interest of stakeholders in this subject from neurology.

The first session was presided over by **Kailash Bhatia (UK)** and **Antonio Federico (Italy)**. A presentation entitled "Rare Neurologic Diseases, a Pandora Box for Neurology and Neurosciences" was introduced by the latter, **Antonio Federico (Italy)**, Professor of Neurology at the Department of Medicine, Surgery, and Neurosciences from the Medical School at the University of Siena (Italy). About 300 million people are affected by a rare disease – 3.5–5.9% of the population – with a majority having genetic pathogenesis and more than half of the cases involving the central or peripheral nervous system and muscles. Prof. Federico firstly discussed what makes a disease rare, namely the standards for classification. Moreover, he went over why rare diseases and orphan therapies lead to many unacceptable inequities in the health systems, from trouble finding experts on the disease, limited availability of therapies, and the issue of pharmacological companies having little interest in developing drugs. Prof. Federico stated the crucial role of neurologists in diagnosing and treating rare diseases in general, as many affections present with neurological manifestations. Furthermore, he discussed the universal challenges faced by those living with a rare disease, mentioning the broad diversity of disorders and relatively common symptoms that can hide the correct diagnosis. He talked about the commonality of misdiagnosis and the variability of symptoms from one patient to another in the context of the same rare disorder. Prof. Federico also tackled in his presentation the importance of research and international collaboration of researchers and clinicians, as well as the involvement of multiple stakeholders, mentioning recent developments in Europe concerning networks aiming to improve the diagnosis and treatment of rare diseases. He further approached the European Reference Networks, especially those for rare diseases, underlining the European Brain Council Research Project – The "Value of Treatment (VOT) for brain disorders". Moreover, Prof. Federico highlighted the World Health Organization's (WHO) highly active endeavour on revising the ICD-10 Diseases of the Nervous System Chapter from a rare disease perspective. Lastly, he mentioned the problem of expensive orphan drugs leading to equity issues in rare diseases. He ended his presentation by stating the necessity for comprehensive global action to improve the quality of diagnosis and care.

**Kailash Bhatia (UK)**, Professor of Clinical Neurology at the Sobell Department of Movement Neuroscience – Institute of Neurology, UCL, in Queen Square, London (UK), discussed the "Rare presentation of Parkinson's diseases and dystonia". Prof. Bhatia covered several case studies on various disorders with variable presentations, from language and calculation difficulties to gait imbalance and cerebellar features of parkinsonism. He showcased imaging clues for different types of parkinsonism, the difficulties in diagnosis, and pointed to different affections, signs, and triggers to be avoided. Rare diseases causing movement disorders are a significant subset of conditions as they represent a substantial proportion of movement disorders. The importance of recognition and awareness of the treatable forms and the treatment implications were also discussed. Finally, Prof. Bhatia highlighted that many rare neurological disorders provide vital insight into the pathophysiology of more common movement disorders.

**Max Hilz (Germany)**, from the Department of Neurology, Icahn School of Medicine at Mount Sinai in New York (USA), discussed, during his presentation "Evaluating autonomic dysfunction in Rare Neurologic Diseases – a diagnostic tool and predictor of increased risk", about hereditary sensory & autonomic neuropathies (HSAN Types I-VIII), the steps in the assessment and consideration for the diseases' epidemiology and their timelines, along with prominent clinical features. Moreover, he presented HSANs types 1, 2, 3, 4, and 5, with insight into the sural nerve pathology, in addition to diagnostic steps to determine the type of HSAN, including history, clinical examination, motor & sensory nerve conduction studies, quantitative sensory testing, assessment of the sudomotor function, of sympathetic or parasympathetic deficits, and many more.

The second session, presided by Marianne de Visser (the Netherlands) and **Michelangelo Mancuso (Italy)**, began by introducing Michelangelo Mancuso (Italy), the Head of the Centre of Neurogenetics and Expertise for Mitochondrial Diseases and Rare Diseases – Department of Clinical and Experimental Medicine at the Neurological Institute, University of Pisa (Italy), with his presentation "Stroke and Rare Neurologic Diseases. An EAN Consensus". Prof. Mancuso discussed the importance of awareness and minimizing diagnostic delays. Moreover, he offered insight into cerebral small vessel diseases (especially the growing area of monogenic cerebral small vessel disease), along with some related issues. Prof. Mancuso followed the Delphi methodology to provide recommendations and proposed "red flag" features suggestive of the diagnosis of monogenic disease and discussed specific recommendations for the particular forms of monogenic cSVD. He further approached some rare diseases, including Fabry disease, CADASIL, and MELAS, and highlighted the EAN management and recommendations, pointing out that the EAN consensus provides a valuable framework to guide diagnosis and management.

**Marianne de Visser (the Netherlands)**, Senior Neurologist at the Amsterdam University Medical Center in Amsterdam (the Netherlands), presented "HyperCKemia: from common to rare". Dr. de Visser discussed asymptomatic and paucisymptomatic hyperCKemia, the most common causes, the steps leading to a differential diagnosis, and the most appropriate diagnostic tools. In addition, she offered a glimpse into variations of the disease in different populations and presented insightful case studies from her practice. One crucial point was the role of complex diagnostic assessment, including detailed family history and ancillary investigations (*e.g*., muscle biopsy, serology, cardiological examination, DNA analysis), offering a lively and engaging presentation with interactive exercises for the public.

**Antonio Federico (Italy)**, Professor of Neurology at the Department of Medicine, Surgery and Neurosciences – Medical School from the University of Siena in Siena (Italy), then discussed "Rare Neurologic Diseases mimicking a multiple sclerosis like phenotype" through the presentation of a comprehensive set of disorders (*e.g*., Adrenoleukodystrophy, Krabbe disease, Paelizaeus-Merzbacker disease, Vacuoliting Leukoencephalopathy, CADASIL, CARASIL and many others), mentioning the McDonald Diagnostic Criteria. For multiple sclerosis (MS), several genetic conditions need to be considered in the differential diagnosis. Prof. Federico offered a complex and all-encompassing view of this large set of disorders and offered the public a glance, through case studies presentation, into the fascinating world of rare neurological disorders.

Session 3 was overseen by Wolfgang Grisold (Austria) and Jean-Marc Burgunder (Switzerland). Firstly, **Davide Pareyson (Italy)**, the Head of Rare Neurodegenerative and Neurometabolic Diseases Unit at the Dept. of Clinical Neurosciences, IRCCS Foundation, Neurological Institute Carlo Besta in Milan (Italy), presented "Clinical and diagnostic approach for diagnosis of rare forms of peripheral neuropathies", discussing the time to consider them as a diagnosis and ways to diagnose these diseases, pinpointing that the most important forms are hereditary and immune-mediated neuropathies. He talked about the role of early diagnosis in detecting diseases and initiating early treatment, and also approached clinical phenotypes for acute neuropathies, chronic sensory-motor demyelinating/axonal, relapsing, pure motor neuropathy, and sensory ataxic or painful neuropathies. Next, Prof. Pareyson presented the steps for diagnosis and the use of next-generation sequencing in certain diseases and hereditary TTR-related amyloid neuropathies in non-endemic countries. Lastly, he discussed Charcot-Marie-Tooth and other chronic immune-mediated or neoplastic neuropathies and underlined the fact that various neuropathies are represented by different phenotypes.

**Wolfgang Grisold (Austria)**, President of the World Federation of Neurology (WFN), presented on the Paraneoplastic Neurological Syndromes, with mentions about the history of the syndromes, different types of most-likely cancers, their mechanisms, therapy, and prognosis. He also discussed the classification of paraneoplastic antibodies based on cancer risk and offered insight into Lambert Eaton Syndrome. One crucial point of his presentation was that the diagnosis of paraneoplastic syndrome is a diagnosis of exclusion.

**Dafin Muresanu (Romania)**, President of the European Federation of NeuroRehabilitation Societies (EFNR) and Chairman at the Department of Neurosciences from Iuliu Hatieganu University of Medicine and Pharmacy in Cluj-Napoca (Romania), offered an inspiring glimpse into the neurorehabilitation for rare neurologic disorders in the context of rare causes of stroke, discussing the concepts of post-lesional brain regulation following two sequences (1) neuroprotection and (2) neurorepair. Prof. Muresanu mentioned that "when neuroprotection is active, neurorecovery is silent" and *vice versa*, showcasing that neurorehabilitation is represented by a mixture of neurorestorative and compensation capacities. He further discussed the intricacies of endogenous defense activity. Neurorehabilitation is a multidisciplinary concept aiming to improve biological function by creating therapeutic learning situations. Prof. Muresanu pinpointed the role of a specialized multidisciplinary neurorehabilitation team (including neurologists, physical therapy specialists, rehabilitation nurses, speech therapists, nutrition specialists, social workers etc). Moreover, the 3 essential components of neurorehabilitation, (1) motor, (2) cognitive, and (3) mood, were discussed, and noteworthy examples of neurorehabilitation effects on different pathologies were presented.

The last session of the day, Session 4, presided by Holm Graessner (Germany) and Maria Judit Molnar (Hungary), began with a presentation of **Maria Judit Molnar (Hungary)**, the Director at the Institute of Genomic Medicine and Rare Disorders Semmelweis University in Budapest (Hungary), on "New therapies for Rare Neurologic Diseases". Prof. Molnar discussed different therapeutic options, their mechanisms on various levels (ARN, DNA, protein level, phenotype) and how pharmacological or surgical approaches can influence the phenotype. She focused on gene therapy, ARN-based methods, and protein modification therapies, mentioning the importance of allogeneic hemopoietic stem transplantation in monogenic neurogenetic disorders. Her presentation showcased general applications of therapeutic gene transfer, gene delivery methods, approved gene therapies, future gene therapeutic approaches in neuromuscular disorders, and valuable lessons from clinical trials. Genetic therapies are increasing extremely fast, opening new options for patients with neurological disorders. Still, there are some limitations, such as the high prices and the ways in which clinicians can support the development of drugs and therapies.

**Jean-Marc Burgunder (Switzerland)**, Professor of Experimental Neurology at the Faculty of Medicine, the University of Bern in Bern (Switzerland), discussed updates in neurogenetics and therapy. First, he offered examples of different diseases (*e.g*., Huntington's disease, hereditary spastic paraplegia, c9orf72 mutation-associated disorders), discussed variations related to the age of onset, and presented an inspiring case study on hereditary spastic paraplegia in twins. The second part of his presentation was structured around the concept of gene therapy, the use of vectors for the restoration of dopamine production and the use of antisense oligonucleotides to modify gene expression. As neurology has a vast number of rare disorders (>7000), with many different genes, pathways, and pathophysiology, there is a dire need to better understand the presented concepts.

**Holm Graessner (Germany)**, Coordinator of the European Reference Network for Rare Neurological Diseases based at the University Hospital in Tubingen (Germany), showcased in his presentation "Undiagnosed Rare Neurologic Diseases and the ERN Role" the European reference networks for complex diseases. One insightful point was that the rareness of disease could refer to the rareness of:

- Patients;
- Experts;
- Care structures of knowledge;
- Therapies.

He discussed the large number of expert centers in 25 EU countries which aim to improve care for patients with neurological diseases (from ataxia to dystonia, paroxysmal disorders, leukoencephalopathy, atypical parkinsonian syndromes, frontotemporal dementia, and many more). They create added value based on the youth partnership plan built to integrate patient representatives and organizations in a structured manner. Moreover, these expert centers monitor the care to sustain improvements, offer e-health for cross-border care, work on expert opinion-based recommendations and provide guidelines.

This awe-inspiring event stands as proof of the significant need for international collaboration and dissemination of knowledge in neurosciences overall, but even more direly for the fascinating yet less addressed topic of rare diseases. As rare neurological diseases can lead to significant inequities in the healthcare system and heavily impact the affected patients, it is paramount to find sustainable solutions, through joint efforts, to address these biases and provide an improved standard of diagnosis and care. It is important to remember that rare diseases not only reflect a reduced incidence but rather the fundamental problems identified by several speakers, namely the lack of specialists, resources directed for the research and development of pharmacological treatments, and the increased chance of misdiagnosis. All of these are completed by a lack of comprehensive understanding of the disease and the best treatment avenues to ensure a high quality of life for the patients.

